# Recommendations for Better Adoption of Medical Photography as a Clinical Tool

**DOI:** 10.2196/36102

**Published:** 2022-07-18

**Authors:** Shannon Wongvibulsin, Kristian Feterik

**Affiliations:** 1 Division of Dermatology Department of Medicine University of California, Los Angeles Los Angeles, CA United States; 2 Department of Internal Medicine University of Pittsburgh Medical Center Pittsburgh, PA United States

**Keywords:** medical photography, photo documentation, EMR, electronic medical record, electronic health record, EHR, interoperability, interoperable, photography, photograph, imaging, image capture, image, image storage, clinical instrument, clinical tool

## Abstract

The use of photography in routine clinical practice has the potential to increase the efficiency of overall patient care as well as improve clinical documentation and provider-to-provider communication. This is particularly important in the setting of provider burnout in the electronic health record era and during the COVID-19 pandemic. Despite the potential of photographs to enhance workflows and patient care, challenges remain that hinder the successful incorporation of medical photography into clinical practice, often because of inconsistent structure and implementation. Our proposed consolidated framework for clinical photography consists of five key aspects: appropriate informed consent; proper preparation and positioning; image acquisition with consideration of the field of view, orientation, focus, resolution, scale, and color calibration; streamlined and secure image storage and documentation; and interoperable file exchange. Overall, this viewpoint is a forward-looking paper on leveraging medical photography as an electronic health record tool for clinical care, research, and education.

## Introduction

Medical photography remains underused as a clinical instrument. Despite the well-acknowledged potential of photographs to improve workflows and patient care, challenges hinder their integration into clinical care [1]. Nevertheless, provider burnout during the electronic health record (EHR) era and COVID-19 pandemic is a growing concern [2] that requires health information technology that supports rather than burdens providers. As the common saying goes, a picture is worth a thousand words; thus, medical photography has the potential to increase efficiency of patient care and improve quality of documentation and provider-to-provider communication. In this viewpoint, we present an overview of the current state of photo documentation, the existing challenges of its adoption and integration into clinical care, and our recommended framework for better use of medical photography as a clinical tool.

## Background: Value of Photo Documentation

Capturing visual representations of patients’ conditions has been essential throughout the history of medicine, from initial documentation through artists’ depictions to the current era of clinical photography using smartphone cameras [3]. In fact, photo documentation has been perceived as less biased than the text record. Providers from diverse fields use photography to record clinical findings. Physicians have reported that photo documentation improves their patient assessment and enhances confidence in clinical decision-making [4]. Patients also perceive the value of photographs and generally approve of the use of clinical images in medical records and coordination of care [4,5], a practice that can help avoid repeated or uncomfortable examinations. Integration of clinical images when communicating with specialists has also been shown to improve the accuracy of the diagnosis [3,6]. Despite all-around interest in photo documentation, there remains a lack of industry consensus for the efficient capture, storage, retrieval, and exchange of digital images in medicine [7-9].

## Current Challenges

Although EHR systems have the capability to store clinical photographs, image capture, documentation, and interoperability are not standardized. Provider workflows range from copying and pasting images into clinical notes to storing images in a dedicated media hub of the EHR. The variability in practices and capability of EHR systems pose challenges in longitudinal patient care, communication between providers, and information sharing with patients. In the longitudinal care of patients, EHR systems usually support trending of vital signs and laboratory values to discern whether a condition remained stable, improved, or worsened. The same functionality is not commonplace with clinical photography, minimizing its utility in tracking conditions over time. Although information exchange has been a long-standing meaningful use objective of the Centers for Medicare & Medicaid Services [10], image file attachments do not reliably display in a consistent manner in the EHR. Some systems strip out file attachments all together. This hinders communication between providers, especially in a medical neighborhood where the patient sees multiple providers using disparate EHRs. Similarly, variable EHR adoption of clinical photography services, transport standards, support of attachments, and inconsistent provider documentation practices complicates the release of images to patients.

## Development of Recommendations

To formulate the following outlined recommendations, we first reviewed prior publications on medical photography. In our review of the literature, we extracted key themes surrounding the challenges in medical photography. Afterward, we enumerated the key aspects for an integrated approach to medical photography based upon both review of the literature and our experiences as medical practitioners working with the EHR for patient care.

## Five Key Steps for an Integrated Approach to Medical Photography

To mitigate ongoing challenges and use medical photography more efficiently, we propose that providers follow these essential steps.

### Obtain Informed Consent

Prior to capturing any images, it is essential to obtain the patient’s informed consent for photography if not already included in the patient’s consent for overall medical care. The process should cover the purpose of clinical photos, access control, identity protection, and image storage. Images intended for publication usually require a separate consent [3,11].

### Prepare and Position

After informed consent, proper preparation and positioning are necessary to obtain high quality photos and minimize any legal risk. Use broad spectrum lighting to avoid shadowing and hot spots. A solid background can improve contrast and prevent artifacts. For image deidentification, move any recognizable information out of the camera’s view to lessen risks for breach of protected health information [11,12].

### Capture Images

Once the area of interest is properly prepared and positioned, verify the patient’s identity and proceed with image capture with the following considerations: field of view (ie, center area of interest), orientation (ie, cephalic orientation), focus (ie, focus on area of interest with camera oriented perpendicular to surface), resolution (ie, when relevant, use the level of resolution that sharply depicts hair follicles or skin markings), scale (ie, place physical scale in area of image capture without obscuring area of interest), and color calibration (ie, ensure that imaging parameters allow for color comparison across images) [12].

### Ensure Streamlined and Secure Image Storage and Documentation

After the images are taken, it is necessary to use a streamlined image archival system linked with the EHR to display the photographs. Ideally, the images should be securely saved directly to the patient’s electronic record from the device capturing the image [11]. This is important because providers can err when taking additional steps during image upload that requires selecting the correct patient and encounter information.

### Establish Image File Exchange Standards to Promote Interoperability

Standards around image file exchange are essential for both provider-to-provider communication and image sharing with patients. Provider-to-provider communication includes two main settings: within the same health system and between separate practices. Typically, image sharing in the same health system is technically and operationally simple. However, for provider communication in unrelated systems, one should consider technical aspects for image transmission. Infrastructure around national image exchanges between systems can draw from examples within radiology and imaging data exchange standards that have been developed for radiographic images [13]. Additionally, implementing transmission standards is essential to ensure data transmission between a trusted and verified sender and receiver for both providers and patients [14].

## Conclusion and Outlook

Numerous areas in medicine can be enhanced through advances in standardization, facilitation, and interoperability of EHR photo documentation, from better tracking of clinical findings over time to improvements in clinical care by improving communication between providers, specialists, and patients. A comprehensive and vast database of deidentified images could allow for development and integration of machine learning algorithms into the EHR. Furthermore, deidentified images can support education of students, trainees, allied practice providers, nurses, and physicians alike. Ultimately, with increasing technological advances in imaging, the possibilities of medical photography to enhance both the patient and provider experience are endless.

## Abbreviations

EHR: electronic health record
